# Willingness to participate in a randomized trial comparing catheters to fistulas for vascular access in incident hemodialysis patients: an international survey of nephrologists

**DOI:** 10.1186/s40697-016-0125-6

**Published:** 2016-07-13

**Authors:** Krishna Poinen, Matthew J. Oliver, Pietro Ravani, Sabine N. Van der Veer, Kitty J. Jager, Wim Van Biesen, Kevan R. Polkinghorne, Aviva Rosenfeld, Adriane M. Lewin, Mandeep Dulai, Robert R. Quinn

**Affiliations:** Internal Medicine, University of Calgary, Calgary, Canada; Sunnybrook Health Sciences Centre, Toronto, Canada; Faculty of Medicine, University of Calgary, Calgary, Canada; Institute of Population Health, Health e-Research Centre, University of Manchester, Manchester, UK; Department Medical Informatics, Academic Medical Center, Amsterdam, Netherlands; UZ Gent, Dienst Nefrologie, Ghent, Belgium; Monash Medical Centre, Melbourne, Australia; Australian and New Zealand Society of Nephrology, Melbourne, Australia; Medicine, University of Calgary, Calgary, Canada; Medicine and Community Health Sciences, University of Calgary, Calgary, Canada; Foothills Medical Centre, Cumming School of Medicine, University of Calgary, 1403 29th Street NW, Calgary, AB T2N 2T9 Canada

**Keywords:** Catheters, CKD, Dialysis, ESRD, Fistulas, Hemodialysis, Vascular access, Chronic renal failure

## Abstract

**Background:**

Current guidelines favor fistulas over catheters as vascular access. Yet, the observational literature comparing fistulas to catheters has important limitations and biases that may be difficult to overcome in the absence of randomization. However, it is not clear if physicians would be willing to participate in a clinical trial comparing fistulas to catheters.

**Objectives:**

We also sought to elicit participants’ opinions on willingness to participate in a future trial regarding catheters and fistulas.

**Design:**

We created a three-part survey consisting of 19 questions. We collected demographic information, respondents’ knowledge of the vascular access literature, appropriateness of current guideline recommendations, and their willingness to participate in a future trial.

**Setting:**

Participants were recruited from Canada, Europe, Australia, and New Zealand.

**Participants:**

Participants include physicians and trainees who are involved in the care of end-stage renal disease patients requiring vascular access.

**Measurements:**

Descriptive statistics were used to describe baseline characteristics of respondents according to geographic location. We used logistic regression to model willingness to participate in a future trial.

**Methods:**

We surveyed nephrologists from Canada, Europe, Australia, and New Zealand to assess their willingness to participate in a randomized trial comparing fistulas to catheters in incident hemodialysis patients.

**Results:**

Our results show that in Canada, 86 % of respondents were willing to participate in a trial (32 % in all patients; 54 % only in patients at high risk of primary failure). In Europe and Australia/New Zealand, the willingness to participate in a trial that included all patients was lower (28 % in Europe; 25 % in Australia/New Zealand), as was a trial that included patients at high risk of primary failure (38 % in Europe; 39 % in Australia/New Zealand). Nephrologists who have been in practice for a few years, saw a larger volume of patients, or self-identified as experts in vascular access literature were more likely to participate in a trial.

**Limitations:**

Survey distribution was limited to vascular access experts in participating European countries and ultimately led to a discrepancy in numbers of European to non-European respondents overall. Canadian views are likely over-represented in the overall outcomes.

**Conclusions:**

Our survey results suggest that nephrologists believe there is equipoise surrounding the optimal vascular access strategy and that a randomized controlled study should be undertaken, but restricted to those individuals with a high risk of primary fistula failure.

**Electronic supplementary material:**

The online version of this article (doi:10.1186/s40697-016-0125-6) contains supplementary material, which is available to authorized users.

## Abrégé

**Mise en contexte:**

À l’heure actuelle, les lignes directrices en matière d’accès vasculaire préconisent l’usage d’une fistule plutôt que d’un cathéter. Or, en consultant la littérature faisant état des études observationnelles comparant l’usage de ces deux types d’accès vasculaire, on note des limites ou un parti-pris qui sont difficilement évitables en absence de randomisation. Néanmoins, il demeure difficile de prévoir si les médecins traitants seraient enclins à participer à des essais cliniques comparatifs de ces deux méthodes.

**Objectifs de l’étude:**

Cette étude avait pour objectif de sonder l’intérêt des participants à prendre part à un essai clinique comparant l’usage des cathéters et des fistules.

**Paramètres de l’étude:**

Pour atteindre cet objectif, nous avons créé un sondage en trois parties constitué de 19 questions. Nous avons colligé les renseignements démographiques des participants, évalué leur connaissance de la littérature et des recommandations actuelles au sujet des accès vasculaires, en plus de sonder leur intérêt à prendre part à un essai clinique à venir.

**Cadre de l’étude:**

Les participants ont été recrutés au Canada, en Europe, en Australie et en Nouvelle-Zélande.

**Participants:**

Des médecins traitants ainsi que des stagiaires en néphrologie, impliqués auprès de patients atteints d’insuffisance rénale terminale et requérant la mise en place d’un accès vasculaire, ont pris part à cette enquête.

**Mesures:**

Des statistiques descriptives ont été utilisées pour définir les caractéristiques démographiques initiales des répondants selon leur situation géographique. Un modèle de régression logistique a été utilisé pour évaluer leur intérêt à participer à un essai clinique.

**Méthodologie:**

Des néphrologues canadiens, australiens, néo-zélandais ou provenant de plusieurs pays d’Europe ont été sondés sur leur intérêt à participer à un essai clinique randomisé comparant l’usage d’une fistule ou d’un cathéter comme accès vasculaire chez les patients nouvellement dialysés.

**Résultats:**

Le sondage a démontré qu’au Canada, 86 % des répondants seraient enclins à prendre part à un essai clinique ; 32 % y feraient participer tous leurs patients sous dialyse alors que 54 % ne le feraient que pour ceux présentant un risque élevé de défaillance primaire de la fistule. Pour le reste des participants, l’intérêt en regard d’un essai clinique ouvert à tous les patients sous dialyse s’est avéré moindre (28 % en Europe, 25 % en Australie et Nouvelle-Zélande), tout comme pour l’idée de n’y inclure que les patients présentant un risque élevé de défaillance primaire de la fistule (38 % pour l’Europe et 39 % pour l’Australie et la Nouvelle-Zélande combinées). Les néphrologues qui pratiquaient depuis plusieurs années, qui suivaient un grand nombre de patients ou qui se définissaient comme des experts de la littérature sur les accès vasculaires étaient plus enclins à participer à des essais cliniques à ce sujet.

**Limites de l’étude:**

La distribution du questionnaire était limitée aux experts de la question de l’accès vasculaire en hémodialyse dans les pays d’Europe participants. Ceci a fait en sorte que le nombre de répondants européens s’est trouvé en déséquilibre en comparaison du nombre de répondants provenant de l’extérieur de l’Europe. De plus, les Canadiens étant surreprésentés parmi les répondants, les résultats obtenus expriment davantage leurs vues que celles des néphrologues provenant d’Australie, de la Nouvelle-Zélande ou des pays d’Europe participants.

**Conclusion:**

Les résultats du sondage suggèrent qu’en général, les néphrologues croient que les deux méthodes se valent au moment de la mise en place d’un accès vasculaire. La plupart sont également d’avis qu’un essai clinique contrôlé et randomisé pour les comparer devrait être réalisé, mais plusieurs croient que celui-ci ne devrait inclure que les patients présentant un risque élevé de défaillance primaire de la fistule.

## What was known before

Fistulas are considered the preferred form of vascular access and are widely promoted by vascular access experts and guideline committees. These recommendations are based on observational data showing an association between fistula use and improved survival, lower procedure rates, and lower costs. There has never been a randomized controlled trial comparing the outcomes of patients treated with fistulas to those treated with catheters, and it was not clear if nephrologists would be willing to participate in a trial.

## What this adds

This survey suggests that nephrologists in Canada, Europe, and Australia/New Zealand would be willing to participate in a future randomized controlled trial comparing outcomes of patients treated with fistulas to those treated with catheters.

## Background

The majority of people with kidney failure are treated with hemodialysis and require access to the bloodstream. While vascular access (“access”) is a lifeline, it is also a key driver of morbidity, mortality, and cost [1]. Arteriovenous fistulas, arteriovenous grafts, and central venous catheters are the three main options for vascular access, but the majority of hemodialysis patients use either a fistula or catheter [2]. Guidelines recommend a fistula for of access based on observational data indicating that use of fistulas is associated with a lower risk of complications and access-related costs and longer patient survival [1, 3–9]. This had led to a number of local, regional, and national initiatives designed to increase the proportion of individuals treated with them [10].

The observational literature comparing fistulas to catheters has important limitations that may have biased previous comparisons and may be difficult to overcome in the absence of randomization [11, 12]. Patients who start dialysis urgently, those who are acutely ill, and those with the highest burden of comorbidity are treated almost exclusively with catheters. Studies including these patients in the catheter group are at high risk of indication bias. Further, most studies did not ascertain access outcomes rigorously or did not report them comprehensively. These studies are at high risk of ascertainment and information bias [13].

The adoption of health-care interventions in the absence of sufficient evidence of safety and efficacy can lead to inefficient use of health-care resources and, potentially, unintended harm to patients. For example, increasing the dose of dialysis delivered to patients was associated with better outcomes in observational studies, but subsequent randomized trials have failed to show benefit [14, 15]. Normalizing hemoglobin levels in patients with kidney disease using erythropoietin-stimulating agents was also felt to be desirable until clinical trials showed that it increased the risk of cardiovascular events [16–21]. However, it is not clear if physicians would be willing to participate in a clinical trial comparing fistulas to catheters given that the observational data have shown a strong and consistent benefit of fistulas, there are incentives for dialysis programs to increase the numbers of patients treated with them in many jurisdictions, and the proportion of patients treated with a fistula is considered a quality measure.

We surveyed nephrologists from Canada, Europe, Australia, and New Zealand to assess their willingness to participate in a randomized trial comparing fistulas to catheters in incident hemodialysis patients. We also sought to elicit physician views about vascular access, their familiarity with the literature, and their opinions regarding current guideline recommendations.

## Methods

### Participants

Physicians and trainees who are involved in the care of end-stage renal disease patients requiring vascular access were eligible to complete the survey. To ensure a broad view of practices, we recruited participants from Canada, Europe, Australia, and New Zealand. This process was facilitated through the Canadian Society of Nephrology (CSN), European Renal Association/European Dialysis and Transplantation Association (ERA/EDTA), and Australia and New Zealand Society of Nephrology (ANZSN). The survey was distributed to all members of the CSN. In Europe, the survey was distributed to individuals with an interest or expertise in vascular access from participating countries, as identified by the ERA/EDTA. Individuals participating in another survey administered by the ERA/EDTA were invited to participate in our survey. The list of physicians who were willing to be contacted was provided to us. In Australia and New Zealand, the survey was distributed to the ANZSN membership. Two reminder emails were sent to increase participation in the survey. The numbers of physicians that the survey was distributed to were not available from the CSN or the ANZSN.

### Survey design

We created a three-part survey consisting of 19 questions that took approximately 10 min to complete. In the first part of the survey, we collected demographic information and information about respondents’ practice environment, as well as the resources available to them. In the second section, we asked about the respondents’ knowledge of the vascular access literature and how it influenced their practices and views. Finally, we asked respondents to comment on the appropriateness of guideline recommendations, whether or not future research was needed in vascular access, and their willingness to participate in a randomized comparison of fistulas to catheters in hemodialysis patients. A copy of the Canadian version of the survey is included as an Additional file 1. The surveys for other jurisdictions did not differ in content, but references to Canada were replaced with Europe and Australia/New Zealand.

### Primary outcome

Our primary outcome was the respondents’ willingness to participate in a randomized trial comparing fistulas to catheters in incident hemodialysis patients. Respondents were also asked to indicate the population that they felt should be entered into a clinical trial (all patients, only patients at high risk of primary fistula failure, or no one, as it was felt to be unethical) and the essential elements of a trial (e.g., minimum follow-up of 3 years, only conducted at sites with a primary fistula failure rate of less than 50 %).

### Statistical analysis

Descriptive statistics were used to describe baseline characteristics of the survey respondents and their programs, according to geographic location. We used logistic regression to model willingness to participate in a randomized trial comparing fistulas to catheters. The model was adjusted for covariates that, a priori, were felt likely to be associated with willingness to participate in a randomized trial based on a review of the literature and clinical intuition. The model included a geographic region (Canada, Europe, Australia/New Zealand), practice setting (urban vs. rural), years in practice (<5, 5–10, 10–15, or >15 years), percentage of time spent in direct patient care (<25, 26–50, 51–75, and >75 %), presence of a nephrology fellowship training program at a primary hospital, risk of primary failure of a fistula at their center (<25, 26–50, 51–75, and >75 %), and familiarity with the vascular access literature (expert vs. non-expert).

Ethical approval was not required for this study.

## Results

A total of 267 nephrologists and nephrology trainees participated in the survey. Of those, 248 respondents fully completed the survey (including the question on their willingness to participate in a trial). The majority of respondents were from Canada (68 %), followed by Australia/New Zealand (21 %) and then Europe (12 %). As the total number of survey recipients from Canada, Europe, and Australia/New Zealand was not disclosed, we were unable to calculate the final response rates.

### Participants

The baseline characteristics of the survey respondents, according to geographic location, are presented in Table 1. The respondents were primarily nephrologists (Canada 98 %, Europe 90.6 %, Australia/New Zealand 100 %) who spent >75 % of their time providing direct patient care (Canada 40.5 %, Europe 48.3 %, Australia/New Zealand 47.1 %) and who worked in urban hospitals (Canada 93.5 %, Europe 93.1 %, Australia/New Zealand 84.3 %).

**Table 1:** Baseline characteristics of survey respondents

	Canada	Europe	Australia/NZ
	*n* (%)	*n* (%)	*n* (%)
Age			
<35	28 (16.7)	3 (10.3)	10 (19.6)
35–50	93 (55.4)	11 (37.9)	30 (58.8)
51–65	39 (23.2)	15 (51.7)	11 (21.6)
>65	8 (4.8)		
Years in practice			
<5	37 (22)	3 (10.3)	18 (35.3)
5 to 10	35 (20.8)	5 (17.2)	10 (19.6)
10 to 15	33 (19.6)	3 (10.3)	7 (13.7)
>15	53 (31.5)	18 (62.1)	12 (23.5)
Percentage of time spent in direct patient care			
<25 %	11 (6.5)	2 (6.9)	3 (5.9)
25–50 %	28 (16.7)	4 (13.8)	10 (19.6)
51–75 %	61 (36.3)	9 (31)	14 (27.5)
>75 %	68 (40.5)	14 (48.3)	24 (47.1)
Primary hospital affiliation is in urban center	157 (93.5)	27 (93.1)	43 (84.3)
Self-identified vascular access expert	26 (15.5)	16 (55.2)	7 (13.7)
Primary hospital has a nephrology fellowship training program	115 (68.5)	23 (79.3)	45 (88.2)
Risk of primary failure of fistulas at local center			
<25 %	52 (31)	20 (69)	30 (58.8)
25–50 %	63 (37.5)	8 (27.6)	8 (15.7)
51–75 %	8 (4.8)	-	1 (2)
Do not know	45 (26.8)	1 (3.4)	12 (23.5)

*NZ* New Zealand

### Infrastructure and support for vascular access creation

Table 2 shows the support available for vascular access creation, according to geographic location. There were significant differences in likelihood of programs having a vascular access coordinator (*p* < 0.01) and dedicated vascular access nurses (*p* < 0.01) among geographic regions, largely driven by the low numbers of programs in Europe that had dedicated vascular access staff and clinics.

**Table 2:** Support available for vascular access creation, by geographic location

	Canada	Europe	Australia/NZ	*p* value
	*n* (%)	*n* (%)	*n* (%)	
	*N* = 168	*N* = 29	*N* = 51	
Vascular access coordinator	149 (88.7)	10 (34.5)	42 (82.4)	<0.01
Dedicated vascular access clinic^a^	109 (64.9)	9 (31.0)	27 (52.9)	<0.01
Surgeon available at center to create arteriovenous accesses	146 (86.9)	22 (75.9)	46 (90.2)	0.18
Type of operator who create(s) vascular accesses at your center:				0.49
Vascular surgeon	152 (90.5)	25 (86.2)	48 (94.1)	
Other (general, urologist, nephrologist, not available)	15 (8.9)	4 (13.8)	3 (5.9)	

*NZ* New Zealand

^a^Staffed by any combination of physicians, surgeons, and vascular access coordinators/nurses with an interest in vascular access

### Willingness to participate in a future RCT

**Fig. 1:**
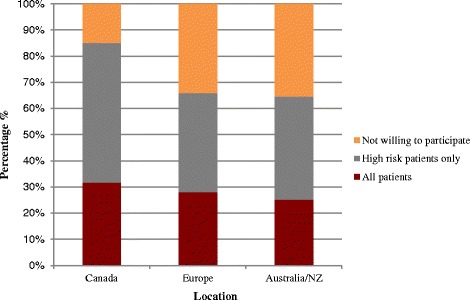
Willingness to participate in a randomized controlled trial comparing fistulas to catheters, by geographic location and patient population. This figure shows the proportion of survey respondents who were willing to participate in a randomized controlled trial comparing catheters to fistulas in incident hemodialysis patients, by geographic location, and according to the patient population included. In Canada, 86 % of respondents were willing to participate in a trial (32 % in all patients; 54 % only in patients at high risk of primary failure). In Europe and Australia/New Zealand, the willingness to participate in a trial that included all incident hemodialysis patients was lower (28 % Europe; 25 % Australia/New Zealand) as was the willingness to participate in a trial that included patients at high risk of primary failure of their fistulas (38 % in Europe; 39 % in Australia/New Zealand). *NZ* New Zealand

Figure 1 illustrates the respondent’s willingness to participate in a randomized controlled trial comparing fistulas to catheters. In total, 79 % of the total respondents agreed that a future trial was needed. While the majority of respondents from Europe (66 %) and Australia/New Zealand (64 %) were supportive of a trial, the proportion in favor was lower than in Canada (86 %).

Thirty-eight percent of nephrologists who were in favor of a trial were supportive of a study in all hemodialysis patients who were candidates for a fistula (37 % in Canada; 45 % in Europe; 40 % in Australia/New Zealand), while 62 % of nephrologists indicated that it should be restricted to individuals at high risk of primary fistula failure (63 % in Canada; 55 % in Europe; 60 % in Australia/New Zealand).

### Variables associated with willingness to participate in a clinical trial

Table 3 shows the variables associated with respondents’ willingness to participate in an RCT. The odds ratio of each variable is adjusted for the potential confounding effect of all the other variables in the model. In a multivariate analysis, participants from Canada were more likely to indicate that they would be willing to participate in a trial compared to respondents from Australia/New Zealand (OR 0.36; 95 % CI 0.16–0.85) or Europe (OR 0.31; 95 % CI 0.11–0.94). Relative to people who had been practicing fewer than 5 years, those in practice for 5–10 years were five times more likely to indicate willingness to participate (OR 5.07; 95 % CI 1.41–18.26). Those respondents that spent greater than 75 % of their time providing direct clinical care were less likely to endorse participation in a trial than those with less than 25 % clinical time (OR 0.86; 95 % CI 0.20–3.63). Participants who self-identified as vascular access experts (OR_expert vs. non-expert_ 2.4; 95 % CI 0.91–6.53), those from urban settings (OR_urban vs. rural_ 1.39; 95 % CI 0.44–4.45), and those who did not know their local risk of primary fistula failure (OR_no knowledge vs. local rate <25%_ 4.08; 95 % CI 1.41–11.8) were also more likely to be willing to participate in a future trial.

**Table 3:** Predictors of respondents’ willingness to participate in a randomized controlled trial

	Adjusted odds ratio	95 % CI	*p* value
Region			
Canada	Reference	–	–
Europe	0.32	0.11–0.91	0.03
Australia/New Zealand	0.37	0.16–0.85	0.02
Number of years licensed and practicing as a nephrologist			
5	Reference	–	–
5 to 10	5.07	1.41–18.26	0.01
10 to 15	1.55	0.54–4.43	0.42
>15	1.20	0.49–2.91	0.69
Percentage of time spent in direct patient care (clinical duties)			
<25 %	Reference	–	–
25–50 %	1.55	0.30–8.02	0.60
51–75 %	0.82	0.19–3.59	0.79
>75 %	0.86	0.20–3.63	0.84
Primary hospital affiliation is urban	1.40	0.44–4.45	0.57
Primary hospital has a nephrology fellowship training program?	0.70	0.29–1.70	0.44
Risk of primary failure for arteriovenous fistulas at your center			
<25 %	Reference	–	–
25–50 %	1.74	0.78–3.91	0.18
51–75 %	0.56	0.12–2.60	0.46
Do not know	4.08	1.41–11.78	0.01
Self-identified vascular access expert	2.44	0.91–6.53	0.08

*CI*, confidence interval, *OR* odds ratio

### Important elements of a clinical trial

Over half of the participants from all countries felt it would be important to have a national funding agency vet the protocol (58 %), while an even greater number suggested that a data safety and monitoring board should provide oversight (77 %). A minimum 3-year follow-up period was felt to be important by the majority of participants (61 %), but participants did not feel that a trial should be limited to centers with a primary failure rate of less than 50 % after fistula creation (34 %) (see Table 4).

**Table 4:** Essential elements of a randomized controlled trial comparing fistulas to catheters in opinion of respondents, by geographic location

	Canada	Europe	Australia/NZ	*p* value
	*n* (%)	*n* (%)	*n* (%)	
	*N* = 168	*N* = 29	*N* = 51	
Protocol should be vetted by a national funding agency	110 (65.5)	11 (37.9)	23 (45.1)	<0.01
Data safety and monitoring board must provide oversight	139 (82.7)	12 (41.4)	40 (78.4)	<0.01
All hemodialysis patients should be studied	50 (29.8)	10 (34.5)	13 (25.5)	0.69
Only certain high-risk patient populations, where the benefit of fistulas is not clear based on observational studies, should be studied	101 (60.1)	14 (48.3)	30 (58.8)	0.49
Only patients who have failed a previous fistula attempt should be studied	12 (7.1)	5 (17.2)	7 (13.7)	0.13
Follow-up must be a minimum of 3 years	105 (62.5)	20 (69)	27 (52.9)	0.31
The study should only be conducted at centers with a primary failure rate of less than 50 % after fistula creation	63 (37.5)	8 (27.6)	15 (29.4)	0.40

## Discussion

Our findings confirm that the majority of nephrologists surveyed from Canada, Europe, and Australia/New Zealand would be willing to participate in a randomized trial comparing fistulas to catheters in incident hemodialysis patients. However, the majority of respondents felt that it should be limited to patients who are at high risk of primary failure of their fistula. Nephrologists from Canada, those who had been in practice for a short duration of time, practiced in an urban setting, and self-identified as vascular access experts were more likely to indicate a willingness to be involved in a trial comparing fistulas to catheters. While fistulas are aggressively promoted, our results would suggest that many practicing nephrologists feel that there is sufficient equipoise to warrant a randomized comparison of catheters and fistulas.

The majority of nephrologists who were in support of a randomized trial comparing catheters to fistulas felt that it should be restricted to individuals with a higher risk of primary failure. Risk factors for failure to mature include age over 65, female gender, the presence of peripheral vascular disease and coronary artery disease, obesity, diabetes, location of the fistula, small-caliber veins, and ethnicity other than Caucasian [22]. A high risk of primary failure may alter the risk-benefit ratio in those undergoing a fistula attempt, as 25–69 % of fistulas created will fail to mature, depending on the patient’s risk profile [22]. If a fistula never works, the patient does not have the opportunity to experience the potential benefits of avoiding a hemodialysis catheter. In addition, failure of fistula maturation has previously been shown to lead to repeated surgical interventions, disruption of the hemodialysis schedule, and patient distress [22]. It is also associated with inefficient use of health-care resources; a trial that tested the benefits of attempting a fistula in this high-risk patient population would inform clinical practice and help providers select the patients most likely to benefit.

Physicians from Canada were more likely to indicate a willingness to participate in a randomized trial comparing fistulas to catheters. While guideline recommendations from Canada, Europe, and Australia/New Zealand consistently recommend fistulas as the preferred form of vascular access for hemodialysis, the prevalent use of fistulas varies. The DOPPS study showed that the prevalence of fistulas in Canada was 50 %, whereas in Europe the range was 57–83 % [23]. In New Zealand, the rates were closer to 60 % [24]. This may be due to differences in the availability of infrastructure or the access to, or organization of services, although our results would suggest that the infrastructure to support fistula creation and maintenance are more developed in Canada than in European centers. It more likely reflects differences in the attitudes and beliefs of health-care providers.

Interestingly, nephrologists who have practiced for 5–10 years were more likely to participate in a trial. Those who were new to practice (<5 years) and those who had been in practice longer (>10 years) were less likely to be willing to participate in a clinical trial. The reasons for this are not clear. New physicians may be more likely to adhere to the principles taught to them during training, whereas clinicians who had been in practice for a long time were more likely ingrained in their practice patterns and less open to the idea of a clinical trial. Individuals who spent more of their time in direct patient care were less likely to want to participate in a trial. The reasons for this are not clear. It may be that physicians with a more clinical focus are more likely to adhere to the teaching they received during their fellowship training and less likely to read the primary literature for themselves or to have had additional training in critical appraisal. This is a potential threat to the feasibility of an RCT, given that the majority of patients are cared for by this group of physicians. Finally, self-identified experts likely have a greater familiarity with the vascular access literature. As a consequence, they may be more aware of the limitations of existing studies—that they are of low quality and prone to bias—and may be more willing to participate in a clinical trial [4]. However, experts are more likely to be involved in the generation of guideline recommendations, and there appears to be discordance between the views expressed in our survey and current guideline recommendations.

Our study has several strengths. First, we recruited respondents from a number of countries in order to explore geographical differences in opinions. Second, this survey is the first, to our knowledge, to ask physicians directly about their willingness to participate in a randomized controlled trial comparing fistulas and catheters. Demonstrating the presence of equipoise is a necessary step in justifying an RCT examining this issue. Our results highlight the discordance between guideline recommendations that consistently recommend fistulas and the views of nephrologists who responded to our survey. Third, our results provide insight into the issues felt to be important considerations in the design of a RCT from the perspective of the practicing nephrologists. This facilitates development of a trial protocol that is feasible and maximizes the likelihood of participation.

Our study also has limitations. The intent of this survey was to capture the perception of nephrologists internationally to inform the development of a future trial. Outside of Canada, we distributed the survey via the European Renal Association (ERA) and the Australian and New Zealand Society for Nephrology. European participants were identified by the ERA and were typically vascular access experts from different countries. This ultimately led to a discrepancy in numbers of European to non-European respondents overall, even though Europe has a much larger population. It was however, felt that European respondents’ views were likely reflective of physician views in their countries of origin, but we are unable to confirm that. As a consequence, Canadian views are likely over-represented in the overall outcomes, but similar results were found for each of the geographic regions examined. The response rates are not calculated because we were unable to obtain an accurate count of the physicians that the survey was distributed to in any region. In addition, we attempted to engage US participants in the survey but were unable to do so. As a consequence, we were unable to gauge physician views and opinions in the USA, which would have been of interest. Our results reflect the views of those physicians who responded to our survey and may not reflect the views of non-respondents or an individual physician’s actual practice. Finally, we have only captured the views of nephrologists. The willingness of patients to be assigned randomly to different vascular access strategies has not been explored and is an important consideration. In a recent study by Manns et al., patients were engaged to identify their priorities for research in kidney disease and identified vascular access as a key area of investigation [25]. Although this does not imply that these patients would participate in an RCT, it does show the awareness regarding the importance of further research in this area from a patient perspective. We are currently testing the feasibility of enrolling patients in a randomized trial that will randomly assign people starting hemodialysis with a catheter to either attempt fistula creation or continued use of a tunneled catheter (NCT02675569).

In summary, the majority of nephrologists we surveyed from Canada, Europe, Australia, and New Zealand would be willing to participate in a randomized controlled trial comparing fistulas to catheters in incident hemodialysis patients. Our survey results suggest that nephrologists believe that the existing evidence forming the base of guideline recommendations about vascular access is of low quality, that there is equipoise surrounding the optimal vascular access strategy for hemodialysis.

## Conclusions

The optimal hemodialysis vascular access is not yet known. The current trend towards fistulas over catheters is based on observational evidence that is limited in scope and prone to bias. A randomized control trial comparing fistulas and catheters would be the most objective and comprehensive study to prove the superiority of one form of access over the other. We surveyed nephrologists from Canada, Europe, Australia, and New Zealand to assess their willingness to participate in the aforementioned trial. Although the number of Canadian respondents was greater than those from other regions, the distribution of responses was similar in all regions. That is, a majority of our respondents were willing to participate in a randomized trial but more so if limited to patients who are at high risk of primary fistula failure.

## Abbreviations

ANZSN, Australia and New Zealand society of nephrology; CSN, Canadian Society of Nephrology; DOPPS, Dialysis Outcomes and Practice Patterns Study; ERA/EDTA, European Renal Association/European Dialysis and Transplantation Association

## Additional file

Additional file 1: Setting priorities for vascular research in Canada. (PDF 256 kb)
