# Supplementary material for: Willingness to participate in a randomized trial comparing catheters to fistulas for vascular access in incident hemodialysis patients: an international survey of nephrologists
Source: Can J Kidney Health Dis. 2016 Jul 13;3:33. doi: 10.1186/s40697-016-0125-6 (PMC4944245; doi:10.1186/s40697-016-0125-6)
Supplement: Additional file 1: — Setting priorities for vascular research in Canada. (PDF 256 kb) [file 40697_2016_125_MOESM1_ESM.pdf]

Setting priorities for vascular research in Canada

Introduction

Vascular access is a key driver of quality of life, morbidity, mortality, and resource use in the hemodialysis patient population. Improving vascular access care has been identified as a strategic priority by numerous organizations in Canada and worldwide.

This brief survey is intended to elicit the views of health care professionals involved in the care of hemodialysis patients about vascular access in Canada. The information collected will be used to focus future studies in this area and to support applications submitted to federal and provincial funding agencies to conduct vascular access research.

Your participation is greatly appreciated. This survey consists of 19 multiple-choice questions that can be completed in approximately 7 minutes and all responses will be kept confidential.

SECTION 1: Information about you, your practice environment, and the resources available to you (10 questions)

SECTION 2: Familiarity with the vascular access literature and how it influences your practice/views (4 questions)

SECTION 3: Guideline recommendations and future research (5 questions)

Setting priorities for vascular access research in Canada

Section 1: The purpose of this section is to collect information about you, your practice environment, and the resources available to you. There are a total of 10 questions in this section.

*1. Which of the following best describes your position?

- ☐ Nephrologist who cares for hemodialysis patients
- ☐ Nephrologist with expertise/interest in vascular access
- ☐ Hemodialysis nurse
- ☐ Vascular access coordinator/nurse
- ☐ Nephrology fellow
- ☐ Surgeon with expertise/interest in vascular access
- ☐ Interventional radiologist with expertise/interest in vascular access
- ☐ Other (please specify):

*2. What is your current age?

- ☐ <35
- ☐ 35-50
- ☐ 51-65
- ☐ >65

Setting priorities for vascular access research in Canada

***3. If you are a nephrologist, how many years have you been licensed and practicing in Canada?**

☐ <5

☐ 5-10

☐ 11-15

☐ >15

☐ I am not a nephrologist

***4. What percentage of your time is spent in direct patient-care (clinical duties)?**

☐ <25%

☐ 25-50%

☐ 51-75%

☐ >75%

Setting priorities for vascular access research in Canada

***5. Is your primary hospital affiliation in an urban or rural setting?**

- [ ] Urban
- [ ] Rural

***6. Do you regularly participate in the care of hemodialysis patients?**

- [ ] Yes
- [ ] No

***7. Does your primary hospital have a nephrology fellowship-training program?**

- [ ] Yes
- [ ] No

Setting priorities for vascular access research in Canada

***8. There are varying definitions of primary failure. For our purposes, we will define it as the inability to ever use a fistula independently for hemodialysis after it is created. What is the risk of primary failure when arterio-venous fistulas are created at your center?**

- ☐ <25%
- ☐ 26-50%
- ☐ 50-75%
- ☐ >75%
- ☐ I don’t know what the risk of primary failure is in our program

***9. Please indicate which of the following resources are available to you at your center (select all that apply):**

- ☐ A surgeon that can create arterio-venous accesses at your hospital
- ☐ A vascular access coordinator
- ☐ A dedicated vascular access clinic staffed by any combination of the following: physician(s), surgeons, vascular access coordinators/nurses with an interest in vascular access

Setting priorities for vascular access research in Canada

***10. Please indicate what type of surgeon creates arterio-venous vascular access for your program (select all that apply):**

- ☐ General surgeon
- ☐ Urologist
- ☐ Vascular surgeon
- ☐ Not available in our program; we refer to another program

Other (please specify):

Setting priorities for vascular access research in Canada

SECTION 2: This section is will collect information about your familiarity with the vascular access literature and how it influences your practice/views. There are 4 questions in this section.

***11. How do you rate your familiarity with the literature comparing outcomes with different forms of vascular access for hemodialysis?**

- ☐ I am an expert in vascular access
- ☐ I have read the seminal articles, but don’t remember the details
- ☐ I have not read the literature myself, but have based my views on expert opinion and practical experience

***12. What is your impression of the level of evidence supporting the arterio-venous fistula (“fistula”) as the preferred form of vascular access?**

- ☐ It is based on numerous, well-conducted randomized controlled trials
- ☐ It is based on a single randomized controlled trial
- ☐ It is based on a large body of observational literature that shows a consistent benefit of fistulas over other forms of vascular access
- ☐ There are conflicting observational studies, but on balance, they suggest that fistulas are the preferred form of vascular access
- ☐ I don’t know what the evidence base is

Setting priorities for vascular access research in Canada

***13. Given that a patient has chosen hemodialysis, which of the following factors do you think should influence decision-making when deciding whether to refer him/her for fistula creation (select all that apply)?**

- ☐ Age
- ☐ History of vascular disease
- ☐ History of diabetes mellitus
- ☐ Obesity
- ☐ Sex (male vs. female)
- ☐ Artery/vein size based on vascular mapping
- ☐ Local primary failure rates for fistula creation
- ☐ Pressure to hit targets for fistula use in your program
- ☐ None of the above; I refer everyone for a fistula

***14. Please indicate which of the following most accurately reflects your views when it comes to referral for fistula creation?**

- ☐ I feel very strongly that fistulas should be attempted in all patients, regardless of age or comorbidities
- ☐ I think the vast majority of patients should be sent for a fistula attempt, but there is the occasional patient that shouldn't be sent for fistula creation due to very advanced age or comorbidities
- ☐ I think the vast majority of patients should be sent for a fistula attempt, but there is the occasional patient that shouldn't be sent for fistula creation due to very advanced age or comorbidities. While this is my opinion, I have reservations about whether or not this approach is appropriate.
- ☐ I think it's appropriate to routinely suggest that patients who are elderly, have vascular disease, or other comorbidities should not go for a fistula creation

Setting priorities for vascular access research in Canada

***15. How comfortable are you with guideline recommendations that state that fistulas are the preferred form of vascular access in all hemodialysis patients?**

- ☐ Fistulas are clearly the best form of vascular access for hemodialysis and guidelines should recommend them for everyone
- ☐ I think guidelines should recommend fistulas, but I would be more comfortable if there was randomized controlled trials supporting them as the preferred form of vascular access for hemodialysis
- ☐ I think the evidence supports fistulas as the preferred form of vascular access, but I'm somewhat uncomfortable making recommendations given the strength of the evidence
- ☐ We should not make treatment recommendations without randomized controlled trials to support fistulas as the preferred form of vascular access

***16. How comfortable would you be if reimbursement to dialysis programs were contingent on meeting targets for fistula use in the hemodialysis population?**

- ☐ I would support that idea
- ☐ I think it would be acceptable if targets took into account differences in patient demographics and comorbidities between programs
- ☐ I would not support that idea in the absence of a randomized controlled trial supporting fistulas as the preferred form of vascular access in hemodialysis patients
- ☐ I would not support that idea, but for other reasons

Setting priorities for vascular access research in Canada

*17. Do you feel that further research is needed in vascular access?

- ☐ No, I am convinced fistulas are the superior form of vascular access and further research will not change my practice
- ☐ Yes, in the absence of randomized trials, further research is needed

*18. Please indicate your willingness to participate in a randomized comparison of fistulas to catheters in hemodialysis patients:

- ☐ I would not participate as I feel that it is unethical to randomize patients to fistulas and catheters
- ☐ I would be willing to participate if the study was restricted to patients with a high risk of primary failure of their fistula (e.g. elderly, vascular disease, diabetics, obese)
- ☐ I would participate in a randomized comparison in any patient population as I feel it is important that we do the study

Setting priorities for vascular access research in Canada

***19. If a randomized controlled trial were designed to compare fistulas to catheters, what do you feel would be the essential elements (select all that apply)?**

- ☐ Protocol should be vetted by a national funding agency [e.g. Canadian Institutes for Health Research (CIHR)]
- ☐ Data safety and monitoring board must provide oversight
- ☐ All hemodialysis patients should be studied
- ☐ Only certain high-risk patient populations, where the benefit of fistulas is not clear based on observational studies, should be studied
- ☐ Only patients who have failed a previous fistula attempt should be studied
- ☐ Follow-up must be a minimum of 3 years
- ☐ The study should only be conducted at centers with a primary failure rate of less than 50% after fistula creation
- ☐ Other (please specify):

20. Please record any additional comments that you feel are relevant in the space below:
